# A Truly Emerging Intestinal Parasitosis

**DOI:** 10.4269/ajtmh.2011.11-0210

**Published:** 2011-09-01

**Authors:** Francisco Cabrera, Hector H. Garcia

**Affiliations:** Department of Surgery, Hospital Militar Central, Lima, Peru; Department of Microbiology, School of Sciences, and Center for Global Health, Tumbes, Universidad Peruana Cayetano Heredia, Lima, Peru; Cysticercosis Unit, National Institute of Neurological Sciences, Lima, Peru; Instituto Peruano de Parasitologia Clinica y Experimental, Lima, Peru

**Figure 1.**
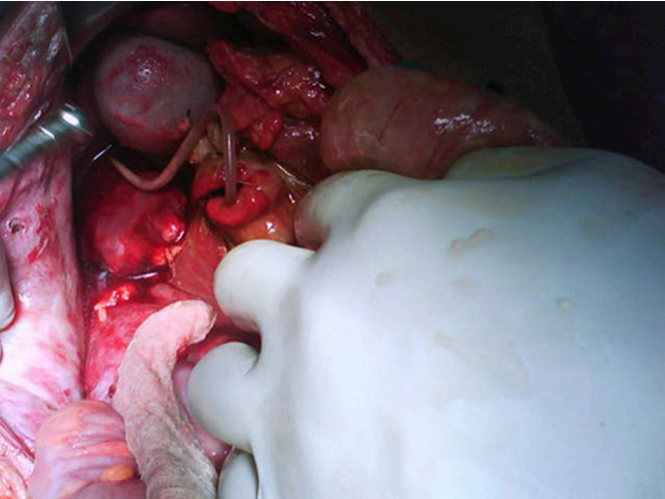
Surgical field of the patient showing a roundworm spontaneously emerging from a wound in the duodenum.

**Figure 2.**
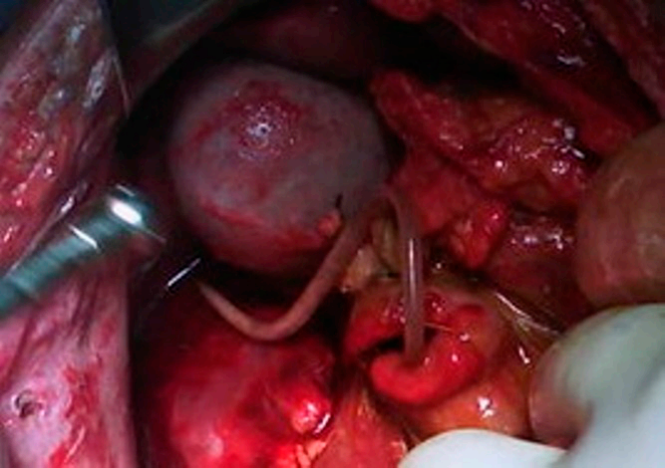
Duodenal area of the patient shown in Figure 1 (original magnification × 2.5).

A 26-year-old soldier from Peru was shot in the right upper back of the abdomen while on routine patrol in a drug-traffic region in the Peruvian highlands. After temporizing exploratory laparotomy in a mobile army surgical hospital, he was flown to a referral center (Trauma-Shock Unit, Central Military Hospital, Lima, Peru) for open surgery. Surgical diagnosis was open abdominal trauma compromising the right kidney, ascending bowel, and a 2-cm duodenal perforation. During cleaning, debriding, and repair of the duodenal perforation (after right nephrectomy and hemicolectomy), a cylindric parasite resembling a large earthworm emerged from the small intestinal wound and swam into the surrounding peritoneal cavity (Figure 1 and Figure 2). The parasite was confirmed to be an adult female *Ascaris lumbricoides.* The patient received antibiotic therapy and 400 mg of albendazole when able to take oral fluids. The patient did well after surgery and was later discharged in good condition.

*Ascaris lumbricoides* is a geohelminth commonly found to infect humans in the developing world.^1^ Adult worms inhabit the small intestine where, if present in large numbers, they can obstruct the intestinal lumen, or actively migrate through the biliary or pancreatic ducts. Although atypical, finding of adult *Ascaris* during intestinal trauma surgery^2^ may not be surprising.
